# Surgical Necrotising Enterocolitis (S-NEC): Where We Stand Today: A Narrative Review

**DOI:** 10.3390/jcm15062236

**Published:** 2026-03-15

**Authors:** Maria Manousi, Dionysios Dellaportas, Konstantinos Nastos, Christina Siouli, Yvelise De Verney, Anastasia Dimopoulou, Nikolaos Zavras

**Affiliations:** 1 First Department of Pediatric Surgery, “Pan. & Aglaia Kyriakou” Children’s Hospital, 11526 Athens, Greece; 2 Third Department of Surgery, Attikon University Hospital, National and Kapodistrian University of Athens, 12462 Athens, Greece; 3 First Department of Pediatric Surgery, “Aghia Sophia” Children’s Hospital, 11527 Athens, Greecenatasa_dimo@hotmail.com (A.D.); 4 First Department of Pediatric Surgery, “IASO” Maternity and Children’s Hospital, 15123 Athens, Greece; 5 Department of Pediatric Surgery, School of Medicine, Attikon University Hospital, National and Kapodistrian University of Athens, 12462 Athens, Greece; nzavras@med.uoa.gr

**Keywords:** necrotising enterocolitis, surgery, laparotomy, laparoscopy, decision-making, clinical, time factors, biomarkers, ultrasonography, artificial intelligence

## Abstract

Surgical necrotising enterocolitis (NEC) continues to carry significant morbidity and mortality in preterm and very-low-birth-weight infants. This review presents up-to-date evidence to guide the shift from medical to surgical treatment and to improve management during and after surgery. Need for surgery is best anticipated through dynamic clinical assessment, supported by laboratory markers of systemic inflammation or ischemia and targeted imaging, while pneumoperitoneum remains the sole absolute indication for immediate intervention. In infants without perforation, the timing of surgery remains challenging: delayed surgery after clinical deterioration worsens long-term outcomes, whereas very early surgery often reflects severe disease leading to greater bowel loss, highlighting the need for carefully timed intervention after brief stabilisation. Laparotomy remains the cornerstone of surgical management, with peritoneal drainage serving as a temporising option for the most unstable infants and laparoscopy emerging as a feasible adjunct. Long-term complications, including strictures, short bowel syndrome, neurodevelopmental impairment, bronchopulmonary dysplasia and severe retinopathy of prematurity highlight the need for better predictive tools, enhanced imaging of bowel viability, and rigorous nutritional support, while long-term quality-of-life outcomes remain insufficiently studied.

## 1. Introduction

Necrotising enterocolitis (NEC) is the most common acquired gastrointestinal emergency in preterm neonates. It is characterised by local and systemic inflammatory responses that may progress rapidly to irreversible intestinal injury, refractory sepsis, and death. Beyond the gut, NEC acts as a systemic disease, precipitating multisystem organ dysfunction, with the brain and lungs being particularly affected [1].

While most cases occur in preterm infants, approximately 10–15% of NEC cases are seen in term neonates [2]. The global incidence of NEC is estimated at approximately 7 per 100 very-low-birth-weight (VLBW) infants in neonatal intensive care units (NICUs), with increasing tendency, likely attributable to advances in neonatal care as well as enhanced detection and reporting practices. However, there is substantial variability in reports from different parts of the world due to differences in healthcare systems and the lack of a clear definition [3].

NEC is difficult to define, as it represents a spectrum of diseases and clinical presentations, from classical “textbook” NEC to fulminant NEC and NEC totalis [1,4]. In addition to conventional NEC in preterm infants, several NEC-like phenotypes have been described, including transfusion-associated NEC (TANEC) [5,6,7], virus-associated NEC [8], NEC associated with congenital heart disease [9], NEC linked to congenital intestinal anomalies such as atresia [10] and Hirschsprung’s disease, and NEC associated with impaired mesenteric flow such as in perinatal asphyxia [4]. Moreover, acquired intestinal injuries that mimic NEC, including focal intestinal perforation (FIP or SIP) and food-protein-induced enterocolitis (FPIES; non-IgE-mediated cow’s milk protein intolerance) [11,12], must be distinguished. Finally, NEC should be differentiated from septic ileus of other aetiologies.

There are eight current definitions of NEC, including (a) Bell staging, (b) modified Bell staging, (c) the Vermont Oxford Network (VON) definition, (d) the United States Centers for Disease Control and Prevention (CDC) definition, (e) the gestational-age-specific case definition of NEC (UK), (f) the two-of-three rule, (g) Stanford, and (h) the International Neonatal Consortium (INC) NEC workgroup definition, with the most widely used being the Bell classification system [13]. Nevertheless, a recent study using machine learning (ML) suggests that non-Bell definitions might be better at diagnosing NEC [14].

Against this background of definitional heterogeneity, a pragmatic distinction is often made based on disease severity and the need for operative management. Surgical necrotising enterocolitis (S-NEC) refers to complicated NEC that progresses to a stage at which operative intervention becomes necessary for survival [15].

NEC is associated with substantial lethality, reported at 23.5% overall and increasing to 40.5% among extremely low-birth-weight infants (<1000 g), with rates reaching as high as 95% in cases of NEC totalis [16,17]. Beyond case fatality, studies examining causes of neonatal death have shown that NEC accounts for approximately one in ten of all neonatal deaths [16]. Lethality following surgical intervention remains high, affecting 34.5% (30.1–39.2%) of neonates undergoing surgery for NEC and 50.9% (38.1–63.5%) of extremely low-birth-weight infants with S-NEC [16]. Long-term morbidity among survivors is also considerable [16,18]. Neurodevelopmental impairment and intestinal failure occur in 24.8% and 15.2% of all children with NEC and rise to 56.8% and 35.3%, respectively, among children with S-NEC [1,16].

It is obvious that NEC and, even more so, S-NEC exert a significant physiological, psychological and financial burden on affected children, their families, and society. Ongoing research continues to explore various aspects, including pathogenesis, prevention, diagnosis, and treatment. In this review, we aim to synthesise the most recent evidence on S-NEC, including risk factors to guide preventive measures, predictive tools, indications and timing of surgery, techniques and outcomes, highlighting current controversies and directions for future research.

## 2. Methodology

A narrative literature review was conducted to summarise current knowledge on the surgical management of NEC in neonates, including pathophysiology, predictors, timing of surgery, operative techniques (laparotomy, peritoneal drainage, and laparoscopy), complications, outcomes, and emerging approaches.

A comprehensive search of PubMed, Google Scholar, and the Cochrane Library was conducted for articles published between January 2019 and February 2025, using combinations of keywords and MeSH terms such as “surgical necrotizing enterocolitis,” “neonatal necrotizing enterocolitis,” “surgical timing,” “laparotomy,” “laparoscopy,” “peritoneal drainage,” “ultrasound,” “artificial intelligence,” “surgical complications,” “long-term outcomes,” and “future directions.” The reference lists of key studies were also manually screened to identify additional relevant publications.

We included English-language, peer-reviewed studies involving neonates with NEC and discussing surgical techniques, timing, pathophysiology and predictors relevant to surgery, or outcomes. Adult-only studies and conference abstracts were excluded; animal studies were eligible when contributing to surgical understanding.

The search yielded several hundred records, of which approximately 209 studies met the criteria and were included in this qualitative synthesis.

## 3. Pathophysiology

### 3.1. Pathophysiology of S-NEC

The pathogenesis of NEC, including its surgical form, remains incompletely understood. It is widely considered to be multifactorial, representing a dynamic balance between intestinal “stressors”, such as prematurity, abnormal gut colonisation, impaired intestinal perfusion, starting even in utero [19], and protective factors, most notably breast milk [20]. Toll-like receptor 4 (TLR4) appears to play a central role in this interplay, as its expression is increased in the intestinal epithelium of preterm infants. Activation of the TLR4 pathway is thought to trigger an exaggerated immune response within the immature intestinal barrier and underdeveloped microvasculature, driven by dysbiosis and formula feeding [19]. This hyperinflammatory cascade leads to enterocyte apoptosis, barrier breakdown, translocation of bacteria and further amplification of inflammation as well as impairment of intestinal regeneration and disruption of the enteric nervous system [21,22]. The transition to S-NEC occurs when these processes culminate in transmural necrosis and ultimately perforation or progressive clinical decline unresponsive to medical therapy, necessitating operative intervention to prevent extensive bowel loss or death.

### 3.2. Risk Factors of S-NEC

#### 3.2.1. Overview of Risk Determinants

Several prenatal, perinatal, and postnatal factors have been identified as key determinants of disease incidence. These risk factors contribute to the overall burden of NEC and, by extension, its severe surgical form (S-NEC). In parallel, emerging research has begun to explore the role of genetic susceptibility, suggesting that inherent molecular and immunological differences may predispose certain infants to the development or progression of NEC [18].

#### 3.2.2. Prenatal Risk Factors

Prenatal risk factors for NEC include intrauterine growth restriction (IUGR) [23,24], placental disease, maternal hypertension and pre-eclampsia [24], gestational diabetes [23,25] and intrahepatic cholestasis of pregnancy (ICP) [25]. Other contributing factors include chorioamnionitis, preterm prelabour rupture of membranes, exposure to amoxicillin–clavulanic acid during pregnancy [23,24], maternal smoking, psychosocial stress [26] and vitamin D deficiency. In contrast, adherence to a Mediterranean diet during pregnancy has been identified as an independent protective factor against NEC [24,27], as has maternal antibiotic use in the third trimester [18,28].

#### 3.2.3. Perinatal Risk Factors

Perinatal risk factors include prematurity and low birth weight [23,24], meconium-stained amniotic fluid (MSAF) [25] and tocolysis with indomethacin, whereas antenatal corticosteroid administration in cases of prelabour rupture of membranes appears to confer a protective effect [23,24]. Although the mode of delivery influences neonatal gut microbiota colonisation and diversity, its direct association with NEC remains unclear.

#### 3.2.4. Postnatal Risk Factors

Postnatal risk factors include early or prolonged exposure to parenteral antibiotics in uninfected preterm infants [18,28,29], use of gastric acid suppressants [29], hyperosmolar enteral feeding, and blood transfusion—particularly in the setting of severe anaemia [18,24,25,30]. Additional risk factors include septicaemia [23,25], congenital heart disease [23,25], patent ductus arteriosus (PDA) [25], pneumonia [23,25] and respiratory distress syndrome [23,25].

Protective postnatal factors include feeding with human milk, which exerts multiple beneficial effects on gut maturation and immunity [23,24,25,29,31], and the use of standardised feeding protocols [29,32]. Nevertheless, the optimal timing for initiation and advancement of enteral feeds remains a subject of debate [32,33,34]. Similarly, oral probiotics may reduce NEC incidence [18,23,25], though controversy persists regarding the ideal probiotic strain, combination, duration of administration and safety [27,35].

#### 3.2.5. Risk Factors Specifically Associated with Surgical NEC (S-NEC)

Several factors have been identified as specifically associated with the development of the surgical form of the disease (S-NEC). Infants with extremely low birth weight, particularly those weighing less than 750 g, and those classified as small for gestational age (SGA) exhibit a markedly increased susceptibility [36,37,38]. Additional perinatal and neonatal predictors include lower gestational age [36,39,40], early onset of NEC [36,39], absence of antenatal corticosteroid exposure [36] and the presence of hypotension or intraventricular haemorrhage (IVH) [38]. A haemodynamically significant patent ductus arteriosus (PDA) has also been consistently implicated as a major risk factor for progression to S-NEC [36,39,41].

Furthermore, antenatal antibiotic exposure [37], administration of nonsteroidal anti-inflammatory drugs (NSAIDs) [42] and the use of arterial or venous umbilical catheters (AUCs and VUCs) [42] have been linked to increased disease severity. The absence of early enteral feeding [40] and the requirement for red blood cell transfusion or cardiovascular support between NEC diagnosis and surgical intervention have also been associated with a higher likelihood of progression to severe disease and poorer clinical outcomes [41].

#### 3.2.6. Integrating Risk Factors into Clinical Assessment

These risk factors should be incorporated into overall assessment to identify neonates at the highest risk of progressing to surgical NEC, in conjunction with clinical, laboratory and imaging predictors and scoring systems that facilitate early recognition and timely management decisions.

## 4. Predicting S-NEC

Predictors of the need for surgery in NEC can be broadly categorised into clinical, laboratory, and imaging parameters (Table 1). These factors support surgical decision-making not as isolated variables but in conjunction with the clinical judgment and experience of the medical team, ideally as part of an individualised treatment plan. Multivariable scoring systems, nomograms, and, more recently, artificial intelligence (AI) approaches such as deep learning (DL) integrate multiple parameters to enhance decision-making regarding surgical intervention (Table 1). Predictors of S-NEC could be regarded as relative indicators within this process, as pneumoperitoneum remains the only absolute indication for surgery to date.

The utility of predictive tools ultimately depends on their diagnostic accuracy, reproducibility, ease of use in routine clinical practice, rapid result availability, and safety for neonatal patients.

### 4.1. Clinical Predictors

Serial physical examination is a timeless and irreplaceable predictive tool for S-NEC. A large multicentre prospective study published by Khalad et al. used a physical examination score comprising seven components: bowel sounds, capillary refill time, abdominal wall erythema, girth, discoloration, induration, and tenderness. It has been shown that a score of ≥3 had a sensitivity of 0.88 and specificity of 0.81 for predicting surgery with AUC of 0.89 [43]. A pooled analysis of physical examinations revealed that abdominal distention had a high sensitivity and abdominal wall erythema had a high specificity in predicting the need of surgery [44].

### 4.2. Laboratory Biomarkers

Common biomarkers that may help predict S-NEC, although not as standalone indicators, primarily reflect systemic inflammation and consequently the severity of intestinal injury. These include elevated plasma C-reactive protein (CRP) [40,45,46,47,48,49,50], C-reactive protein-to-albumin ratio [51,52], high plasma procalcitonin [45,49,53], acidosis [36,39,50,54], thrombocytopenia [46,50], high mean platelet volume (MPV) and platelet distribution width (PDW) [55], platelet-to-lymphocyte ratio (PLR) [45], lymphopenia [46,47,50], neutropenia [45,54,56], coagulation disorder [40,57], hyponatremia [40,50], and hypoalbuminemia [48]. Bianchi notes that metabolic acidosis, falling platelet counts, and escalating inotropic support to maintain circulation and renal function signal profound clinical deterioration [66]. Such findings reflect severe sepsis and represent very late stages of S-NEC, indicating a major intra-abdominal catastrophe requiring immediate surgical intervention for survival. At this point, the window for bowel-sparing surgery has likely been lost [66]. Intestinal fatty-acid-binding protein (i-FABP), detectable in urine or plasma and specific to enterocytes affected by ischemia, inflammation, or mucosal injury, is highly specific [49,58] but currently impractical for routine use due to time-consuming analysis and limited laboratory availability.

### 4.3. Diagnostic Paracentesis

The role of diagnostic paracentesis in guiding surgical decision-making has been a subject of debate. Older reports from nearly 50 years ago highlighted its value in detecting bowel necrosis and perforation [59,60]. A more recent study found that a positive paracentesis (defined by Kosloske’s criteria as ≥0.5 mL of cloudy fluid or clear fluid with a positive Gram stain), when combined with thrombocytopenia, neutropenia, and hyponatremia, was a strong predictor of better surgical outcomes. However, the authors noted that in their own institution, the procedure was performed infrequently due to concerns about potential complications [17]. In contrast, a national survey conducted in China in 2022 reported that 76.3% of paediatric surgeons routinely perform diagnostic peritoneal aspiration [61], highlighting significant regional variation in practice.

The evidence base for paracentesis has recently been strengthened by a randomised controlled trial published in 2026 [70]. This study of 117 preterm neonates with S-NEC compared traditional Bell’s criteria with a strategy combining metabolic disorder components (MD7) and positive paracentesis. The paracentesis-guided approach was associated with significantly lower mortality (32.1% vs. 64.7%; *p* < 0.05), earlier surgery, and better outcomes for perforation, intestinal necrosis, and reoperation. No complications were attributed to the procedure itself.

### 4.4. Imaging Predictors

#### 4.4.1. Abdominal Radiography

Abdominal X-ray remains the gold-standard imaging modality for assessing the need for surgery in NEC and is incorporated into Bell’s staging system. Pneumoperitoneum on abdominal X-ray remains the only absolute indicator for surgical intervention in NEC to date. Subdiaphragmatic air is the most obvious imaging feature on erect abdominal X-ray, whereas by supine X-ray the sign of lucency over the liver shadow, liver falciform ligament sign, Rigler sign, football sign and triangle sign confirm the perforation in S-NEC with 86.73% sensitivity and 100% specificity [62]. In addition, portal venous gas and fixed intestinal loops on plain radiographs demonstrate high specificity but low sensitivity for predicting the need for surgery [44]. Furthermore, an increased bowel loop diameter relative to vertebral landmarks has been associated with S-NEC [63]. Major limitations of plain radiography include exposure to ionising radiation and failure to radiographically detect free peritoneal gas in nearly half of perforated NEC cases [64].

#### 4.4.2. Abdominal Ultrasound

Although not integrated in formal NEC guidelines, there is increasing clinical adoption of standardised ultrasound protocols as an adjunct [71]. Interestingly, in China a total of 81.3% of surgeons reported the use of ultrasonography according to a recent national survey [61]. Sonographic signs in advanced stages or complicated NEC needing surgery include thinned bowel wall (<1.5 mm) [65,72], diminished to absent peristalsis [65,72], reduced intestinal blood signal [72], complex ascites and pneumoperitoneum [65]. Absent perfusion, marked bowel wall thinning, and loss of peristalsis are further regarded as late indicators warranting operative intervention [66]. US parameters predicting intestinal resection in NEC include thick-to-thin transformation, absence of bowel wall perfusion and portal venous gas [67]. Notably, in a study by Kim et al., it was shown that 25% of infants with radiographic pneumoperitoneum studied did not show US findings indicative of perforation or S-NEC, suggesting that both abdominal radiographs and US may be needed to diagnose S-NEC [64].

### 4.5. Scoring Systems and Nomograms

Any single clinical, laboratory or imaging parameter has only limited capacity to predict S-NEC. The prediction accuracy seems to be improved through scoring systems and nomograms combining those parameters [73,74,75,76,77,78,79,80].

Among these scoring systems, metabolic derangement 7 score (MD7) has been meta-analysed as the most frequently used. It includes seven criteria (positive blood culture, acidosis, bandemia, hyponatremia, thrombocytopenia, hypotension, and neutropenia), which score 1 point each, and it is designed for infants without pneumoperitoneum. MD7 score was found to be effective in identifying S-NEC with a specificity and sensitivity both in excess of 70% using a threshold ≥ 3 [58].

Notably, Li et al. developed a scoring system for predicting bowel resection incorporating the following criteria: birth weight, hypotension, neutropenia, pneumoperitoneum, acidosis and intestinal wall thickness. The scores in this system ranged from 0 to 18.1, and scores of 7 and 13 were considered as cut-off points, which could discriminate infants with bowel loss [81].

### 4.6. Artificial Intelligence Predictors

Artificial intelligence (AI) and machine learning (ML) currently play an emerging, adjunctive role in surgical decision-making for NEC. The ResNet18 model, constructed using bedside chest and abdominal imaging, was shown to effectively predict S-NEC [68]. A nationwide cohort study by Kim et al. analysed the perinatal factors of VLBW infants acquired within 7 days of birth and used ML-based analysis, which successfully identified which infants with NEC are vulnerable to clinical deterioration and at high risk for surgical intervention [82]. Moreover Jin et al. developed a CatBoost algorithm to identify the need for surgery in infants with necrotising enterocolitis [69]. Despite these advances, AI is still constrained by data quality and lacks the reliability for independent surgical decision-making [83].

### 4.7. Integrating Predictors into Clinical Decision-Making

Close monitoring remains central to the management of NEC. This includes frequent clinical, laboratory, and imaging reassessment. Peritoneal paracentesis may represent a useful adjunct in selected cases, although its use varies across centres. Structured multidisciplinary evaluation and effective shift handovers can reduce subjectivity and support the timely identification of clinical deterioration requiring surgical intervention.

## 5. Timing in S-NEC

### 5.1. Initial Medical Management and Goals of Stabilisation

Following a diagnosis of NEC, initial management is conservative and typically entails cessation of enteral nutrition, gastric decompression, administration of broad-spectrum antibiotics, and provision of parenteral nutrition, accompanied by early surgical consultation. The overarching aim is haemodynamic and metabolic stabilisation of the infant, irrespective of whether the condition ultimately resolves without operative intervention or progresses to require surgery. The duration of medical therapy is determined by the infant’s clinical trajectory and evolving surgical indications. Definitive evidence of recovery is demonstrated by the infant’s capacity to tolerate the cautious reintroduction and advancement of enteral feeding.

### 5.2. Indicators for Urgent Surgical Reassessment

In contrast, clinical deterioration despite optimal medical therapy warrants urgent surgical reassessment. The presence of free intraperitoneal air, indicating intestinal perforation, is an absolute indication for operative intervention and requires surgery within a narrow and well-defined time window. However, in the absence of pneumoperitoneum, decision-making becomes more challenging. The optimal timing for surgical intervention in radiologically non-evident perforation remains poorly defined [84,85,86,87,88].

### 5.3. Historical Perspective and Evolving Concepts of Surgical Timing

Historically, surgical treatment was not prioritised, as clinicians preferred to allow the diseased bowel to demarcate, minimising the risk of unnecessary resection of potentially viable tissue. A recent study also proposes deferred surgical treatment after the acute episode as a safer option which enables shorter resections and more favourable postoperative outcomes [89]. However, most recent studies increasingly explore the relationship between surgical timing and long-term outcomes [84,85,90].

### 5.4. Evidence on Delayed Versus Earlier Intervention

#### 5.4.1. Delayed Intervention and Associated Outcomes

Bethell et al. identified a significant association between surgical indication, timing, and outcomes in NEC. Infants who underwent surgery for failed medical treatment had a longer delay to intervention compared to those operated on for perforation or a suspected necrotic bowel. This delay correlated with poorer outcomes, with these infants experiencing over four times the odds of prolonged PN dependence or death at 28 days compared to those who underwent surgery for perforation [86].

#### 5.4.2. Risks of Very Early Intervention

Conversely, Rauh et al. found that infants undergoing surgery within 48 h of NEC diagnosis had greater bowel resection and higher mortality, particularly when excluding cases with pneumoperitoneum. They suggested that a brief period of medical stabilisation before surgery might be beneficial, though further studies are needed to determine optimal timing [87].

#### 5.4.3. Morbidity Associated with Delayed Surgery

Garg et al. recently reported that delayed surgery (>48 h) in preterm infants increased perioperative complications, such as acute kidney injury (AKI), longer PN needs, adhesions, and cholestasis, without affecting mortality or systemic morbidities such as bronchopulmonary dysplasia (BPD) or white matter injury. Earlier surgery may lessen some complications, but further multicentre studies are required [84].

### 5.5. Surgical Timing in NEC Totalis

Magdits et al. reported a case series of three infants with NEC totalis who underwent surgical intervention at markedly different intervals after diagnosis: 8 weeks, 12 days, and 24 h. Only the infant who was operated on within 24 h survived, ultimately requiring a jejunostomy and developing short bowel syndrome. This small series underscores the extremely high mortality associated with NEC totalis, regardless of the timing of surgical intervention. Nonetheless, despite the study’s limited numbers, and in light of contemporary improvements in short bowel syndrome management, the authors propose that operative timing might still play a meaningful role in determining outcomes [88].

### 5.6. Close Monitoring as a Framework for Surgical Timing

Given the conflicting evidence on optimal surgical timing [84,87,91], researchers proposed frequent clinical and Doppler-sonographic assessments to guide intervention [66]. In this model, surgical timing is determined by evolving clinical trajectory rather than static factors like gestational age. Key indications for surgery include worsening condition, tender abdomen, palpable bowel loops, progressive distension, and ultrasound findings of thickened bowel loops or free fluid with debris. Abdominal drainage and peritoneal lavage may help stabilise selected infants prior to laparotomy [66].

### 5.7. Synthesis: Balancing Risks of Delay and Premature Intervention

**Figure 1:**
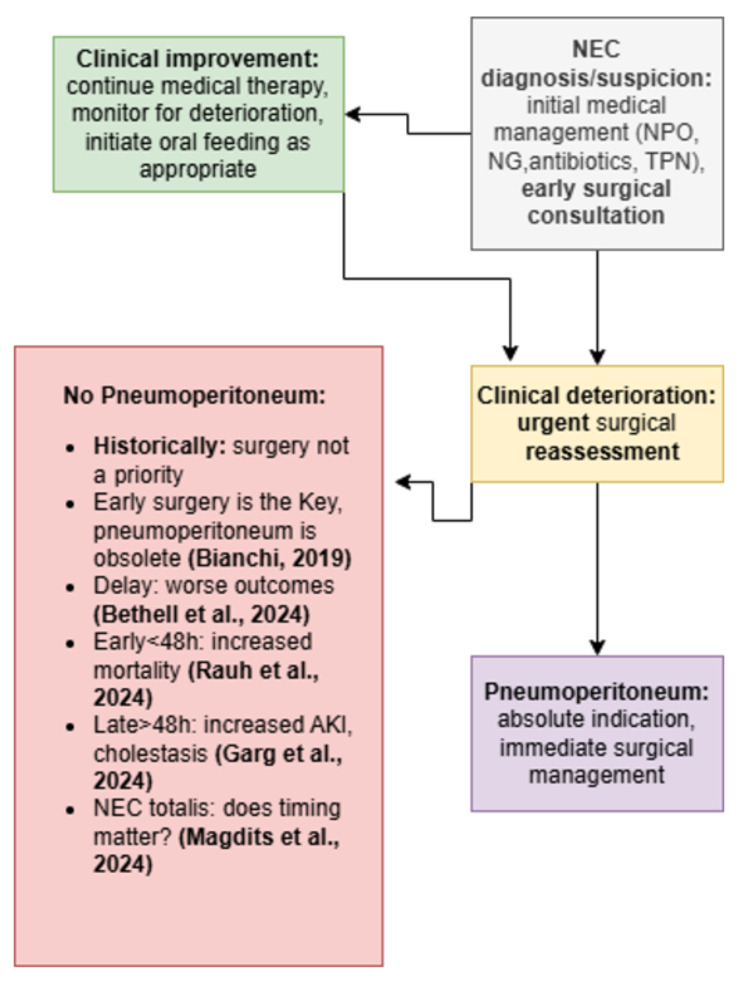
Timeline of surgical decision-making in NEC. Abbreviations: NPO, nil per os (nothing by mouth); NG, nasogastric; TPN, total parenteral nutrition; AKI, acute kidney injury.

Together, these findings reflect a complex and evolving landscape: while excessive delay may worsen outcomes, overly early surgery may increase morbidity (Figure 1). The close monitoring framework discussed above supports earlier selective intervention, guided by clinical deterioration rather than traditional radiologic markers, to avoid postponing necessary treatment while the infant remains physiologically stable [66].

## 6. Management Practices in S-NEC

Debate persists not only regarding the optimal timing but also the surgical approach and technique, driven by disease heterogeneity, the perceived surgical tolerance of preterm neonates, differing expertise, and the need for balance between protocolised and individualised care.

**Figure 2:**
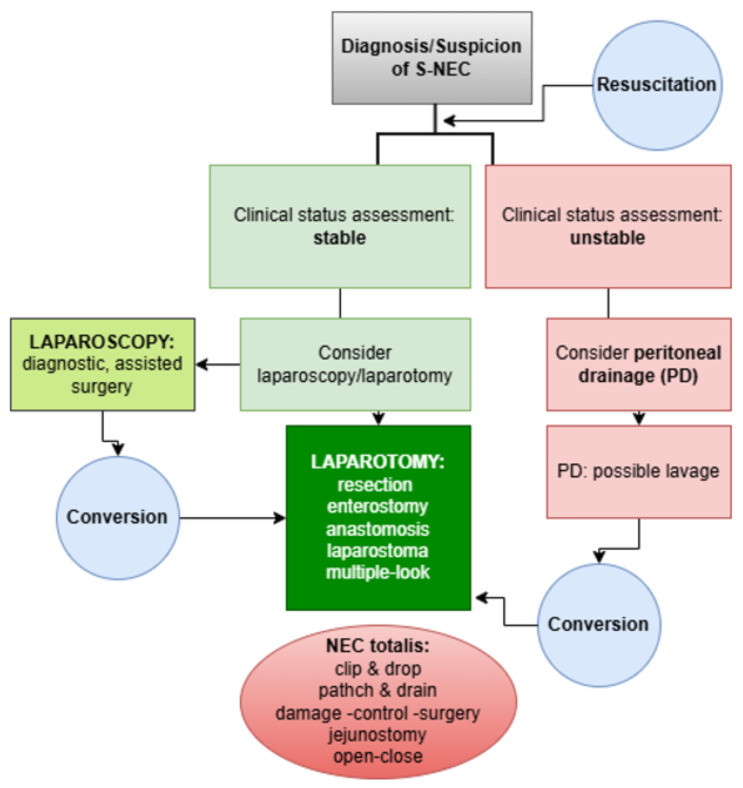
Flowchart for the surgical management of NEC.

There are three main surgical approaches: laparotomy with abdominal exploration—which remains the prevailing strategy and may include a second/multiple-look procedure or laparostomy—primary peritoneal drainage (PPD) with or without subsequent laparotomy, with or without peritoneal lavage [20,90], and laparoscopy, whose role is increasingly being investigated [92] (Figure 2).

### 6.1. Primary Peritoneal Drainage (PPD)

There are various approaches to primary peritoneal drainage (PPD), including the use of a single stab incision or two incisions, typically placed in the right or left iliac fossa or at the umbilicus. Surgeons also differ in whether they irrigate the peritoneal cavity, and in the choice of drain type and timing of removal [90]. The main advantage of PPD is that it is a bedside procedure performed under local anaesthesia, allowing rapid, minimally invasive intervention in critically ill neonates and offering logistical advantages in resource-limited settings [93]. Its major limitation is that most infants undergoing PPD will ultimately require a laparotomy [94]. Evidence remains mixed: a retrospective cohort study by Shen et al. in VLBW infants with Bell’s stage II NEC reported that PPD significantly improved clinical status, reduced length of stay, and appeared to reduce strictures and perforations compared with conservative management. Cure rates between the PPD and laparotomy groups were similar [93]. A recent Cochrane review (2025) concluded that PPD likely results in no difference in mortality or overall neurodevelopmental outcomes at 18–24 months compared with laparotomy in preterm VLBW infants with S-NEC or SIP but may increase the risk of moderate-to-severe cerebral palsy and escalation to laparotomy [94]. In line with the guidelines of the European Reference Network for Inherited and Congenital Anomalies, families should be counselled that many infants treated with PPD may not improve—and may deteriorate—ultimately requiring laparotomy [15].

### 6.2. Laparotomy

Laparotomy remains a definitive surgical option in severe NEC. A multicentre randomised controlled trial by Blakely et al. demonstrated reduced mortality and neurodevelopmental impairment at 18–22 months in ELBW infants with NEC who underwent laparotomy compared with those initially managed with peritoneal drainage, especially in cases characterised by widespread bowel injury rather than localised pathology typical of SIP [95]. Conversely, a large systematic review and meta-analysis by Li et al. that included three RCTs and 49 observational studies (19,940 preterm infants with S-NEC or SIP) found no difference in survival when analyses were restricted to high-quality RCTs. Although pooled observational data suggested lower survival among infants treated with PPD, this likely reflects selection bias and confounding by illness severity. Interpretation of these findings is further limited by the small number of robust trials, substantial heterogeneity, and the combined analysis of NEC and SIP, which complicates direct comparisons between treatment strategies [96,97].

### 6.3. Laparoscopy

The role of laparoscopy in the management of NEC is receiving increasing attention. In theory, it may be particularly valuable during the early stages of the disease, when clinical and radiological findings are equivocal, as it allows for the exclusion or confirmation of NEC and enables direct visualisation of bowel viability to assess disease extent and early detection of perforation. From this perspective, laparoscopy has a role in early S-NEC when distension and inflammation are limited, permitting lavage, drainage, limited resection of non-viable bowel, and bowel decompression with the option of a low ileostomy via the umbilical port. In more extensive disease or when laparoscopic access is inadequate, open laparotomy is favoured for thorough peritoneal cleansing and full bowel inspection [66]. Numanoglu et al. demonstrated that laparoscopy facilitates early identification of perforation and necrosis, although definitive surgical management in their series ultimately required laparotomy [98]. This concept is supported by a recent experimental study in neonatal piglets with NEC [99].

Despite its theoretical advantages, laparoscopy has not achieved broad international adoption in the management of NEC. Conversely, data from a nationwide survey in China showed that 40.2% of paediatric surgeons employed laparoscopy for diagnostic or therapeutic purposes, indicating a significantly higher level of implementation [61].

More recently, Montalva et al. renewed interest in laparoscopy by examining the impact of carbon dioxide insufflation itself. Their findings suggested that early laparoscopic-assisted surgery, compared with open laparotomy, was associated with reduced postoperative inflammation and a lower incidence of intestinal strictures in infants with NEC [92]. Although it remains unclear whether these benefits were due to carbon dioxide pneumoperitoneum or simply to earlier surgical intervention [100], as timing may have influenced the observed outcomes, and despite the high conversion rate to laparotomy (90%), the study provides important evidence that laparoscopy is both safe and feasible in this setting [101]. The question of whether laparoscopy exerts a direct, time-independent effect on the inflammatory response remains unresolved.

It is noteworthy however that earlier studies have consistently demonstrated favourable effects of laparoscopy on the systemic inflammatory response, bacterial translocation, and outcomes in bacterial peritonitis [102]. These observations have been further supported by several animal studies [103,104], reinforcing the potential physiological advantages of minimally invasive approaches in intra-abdominal infections. Such information may hold relevance for NEC, although generating robust evidence in this critically ill population remains challenging.

### 6.4. Intraoperative Assessment of Bowel Viability

Independent of the chosen surgical approach—whether laparotomy or laparoscopy—the primary objective of operative management in NEC remains the control of sepsis while minimising operative time and preserving bowel length [20].

Laparotomy and laparoscopy both require the ability to visually assess bowel viability intraoperatively. While surgical experience remains fundamental, the use of indocyanine green (ICG)-enhanced fluorescence is increasingly being investigated in paediatric gastrointestinal surgery for evaluating bowel perfusion to guide resection or assess anastomosis [98,105,106,107,108].

A recent prospective clinical study reported encouraging findings. In all patients, satisfactory fluorescence was obtained in under one minute using a mean ICG dose of 0.14 mg/kg, and no ICG-related adverse events were observed. Notably, the investigators identified a significant discrepancy between the estimated resection margins based on conventional intraoperative assessment and those suggested by ICG fluorescence angiography (mean difference 2.5 cm, SD 6.2; *p* = 0.016). In most cases, ICG-FA indicated that a greater length of viable bowel could be preserved [109].

However, the interpretation remains largely subjective, as no standardised perfusion thresholds have been established for paediatric patients [105,109]. Furthermore, dosing recommendations for children vary according to indication and age. Particularly, ICG use in neonates is still limited [110]. An additional disadvantage is the restricted availability of the required imaging equipment, largely due to its cost.

### 6.5. Resection and Reconstruction Strategies in S-NEC

#### 6.5.1. Determinants of Operative Strategy

The choice of operative technique is further determined by the extent of disease, the physiological status of the neonate, as well as the surgeon’s and centre’s experience.

#### 6.5.2. Intraoperative ABC Classification System

Shang et al. introduced an intraoperative ABC classification system for surgical NEC, grounded in damage-control surgery principles [111]. The approach shifts emphasis away from routine extensive resection of all compromised bowel toward rapid decompression to prevent retrograde progression of ischemia. The rationale is that decompression may salvage ischemic but not yet frankly necrotic tissue, limiting the extent of resection. This does not imply that clearly necrotic tissue should be left in situ; as discussed later, once necrosis is established, prompt resection remains essential.

The classification also links anatomical involvement to prognosis: outcomes are more favourable in pure colonic NEC (Type B) than isolated small-bowel disease (Type A). In combined small-bowel and colonic involvement (Type C), the authors often favoured creating a small-bowel stoma without resecting the necrotic colon to limit operative trauma. In multifocal disease, they advise against preserving very short segments of marginally viable bowel when the remaining length is sufficient, as this prolongs surgery without clinical benefit [111].

#### 6.5.3. Extent of Resection: Complete Resection vs. Leaving Diseased Bowel In Situ

Considerable debate persists regarding whether complete resection of necrotic bowel—aimed at controlling sepsis—justifies the increased risk of short bowel syndrome. In a recent study, Pardy et al. compared two operative strategies: complete resection versus leaving diseased bowel in situ at the initial laparotomy. Their findings indicated higher survival in the complete-resection group, while rates of eventual enteral autonomy were comparable between the two approaches. Total NEC cases were excluded from the analysis. In this cohort of 44 patients, an enterostomy was performed in 36 infants, 2 underwent primary anastomosis, and 4 were managed with a clip-and-drop technique. It is important to recognise that surgical decision-making is influenced by haemodynamic stability and patient characteristics; smaller and more premature neonates are often managed with a defunctioning stoma without resection, and this subgroup is known to have intrinsically higher mortality from NEC [112].

#### 6.5.4. Stoma Formation: Benefits and Limitations in S-NEC

Another key topic in the surgical management of NEC concerns the choice between primary anastomosis and stoma formation. In addition to the factors discussed above, stoma formation is often chosen due to concerns about ongoing NEC activity despite surgical intervention. Consequently, clinicians may favour stoma creation over primary anastomosis when disease severity is uncertain or the risk of progression is high [113]. Distal stoma refeeding also permits controlled enteral support while limiting complications associated with prolonged total parenteral nutrition, particularly in neonates at high risk of feeding intolerance [114]. Nevertheless, these concerns may be overestimated in many cases in which primary anastomosis could be safely performed.

It is well recognised that neonates with NEC who undergo enterostomy, in addition to facing stoma-related complications, such as stenosis, ischemia, dehiscence, retraction, prolapse and fistulation [115], are at heightened risk of significant growth impairment [116]. Subsequent stoma closure has been associated with improved head-circumference growth in very preterm infants [117].

#### 6.5.5. International Variability in Surgical Practice

An international comparison of NEC management practices in the Netherlands and Finland, published in 2025, demonstrated substantial variation: Finnish centres most frequently employed ostomy creation, whereas Dutch centres more commonly performed primary anastomosis [118]. In Sweden, resection of necrotic bowel with stoma formation is the dominating surgical practice [119]. In the United States, Goldfarb et al. reported findings from a large, geographically diverse cohort of infants treated across children’s hospitals, showing that only 15.8% underwent primary anastomosis. Importantly, rates of wound and infectious complications, duration of parenteral nutrition, and length of hospital stay were comparable between infants managed with primary anastomosis and those undergoing an enterostomy [113].

#### 6.5.6. Evidence Supporting Primary Anastomosis

Building on these observations, the STAT trial, a multicentre randomised controlled study conducted across 12 international centres, demonstrated that primary anastomosis should be performed when no disease is present distal to the resected bowel segment, as this approach is associated with improved postoperative recovery [120]. This recommendation is further supported by the guidelines of the European Reference Network for Congenital and Inherited Anomalies [15].

In extremely low-birth-weight infants with perforated NEC (<1000 g), Alzamrooni et al. found that primary anastomosis was associated with shorter hospital stay and earlier enteral autonomy, with mortality comparable to stoma formation. Outcomes were similar even among micro-preterm infants (<750 g), indicating that extreme prematurity alone should not preclude primary anastomosis [121].

Additionally, a recent systematic review and meta-analysis by Alansari et al. reported that, in selected neonates with NEC, primary anastomosis is associated with lower mortality and complication rates comparable to those observed with stoma formation [122]. However, interpretation of these findings is limited by the retrospective design of all included studies. The higher morbidity and mortality reported in the stoma group may reflect greater underlying disease severity rather than the effect of the surgical approach itself [122].

#### 6.5.7. Anastomotic Complications and Risk Factors

Scepticism persists regarding the selection criteria for primary anastomosis, with concerns that overly broad indications may lead to excessive enthusiasm and increase the risk of anastomotic or parenteral-nutrition-related complications [123].

The severity of preoperative illness remains a critical determinant in surgical decision-making, as haemodynamically unstable infants are generally considered unsuitable candidates for primary anastomosis [121].

## 7. Management Practices for Multifocal NEC and NEC Totalis

### 7.1. Definition and Scope of Multifocal Disease and NEC Totalis

One of the greatest challenges in the surgical management of NEC is the treatment of extensive multifocal disease, including NEC totalis or near-totalis with <30 cm of remaining bowel, although no formal definition exists and considerable variability and subjectivity persist in how these conditions are described [15,90,111,124].

### 7.2. Ethical Considerations and Prognostic Uncertainty

These cases present a profound ethical dilemma, as clinicians must determine whether aggressive medical and surgical intervention is appropriate or whether palliative care should be pursued [90]. Evidence suggests, however, that infants with post-NEC intestinal failure may have a greater potential for weaning from parenteral nutrition than those with short bowel syndrome from other causes [15].

### 7.3. Guidance from the European Reference Network

The European Reference Network’s good practice statement emphasises that management decisions should rest with the treating team but must not rely solely on residual bowel length, as long-term outcomes, including neurodevelopmental trajectories in these preterm infants, remain uncertain [15].

### 7.4. Surgical Strategies in Multifocal NEC and NEC Totalis

#### 7.4.1. Patch, Drain, and Wait Technique

Many different surgical approaches have been described. In 1989, Moore et al. proposed the ‘patch, drain and wait technique’, which consists of covering the perforation with sutures, positioning two drains and then waiting with long-term parenteral nutrition [124].

#### 7.4.2. Clip-and-Drop Technique

The ‘clip-and-drop’ technique offers an alternative approach with better clearance of the abdomen: clearly necrotic or perforated segments are resected, the bowel ends are temporarily closed (often with non-absorbable clips), and the abdomen is closed without creating an anastomosis or stoma. A planned second-look laparotomy 24–72 h later allows reassessment of bowel viability and, if feasible, definitive reconstruction or stoma formation [125]. There is limited published evidence on clinical outcomes following this intervention. Ron et al. reported a two-centre experience demonstrating that, despite the need for multiple procedures, approximately half of the infants in this high-risk group survived and ultimately achieved full enteral feeding [126].

#### 7.4.3. Damage-Control Surgery

Damage-control surgery, originally developed in the context of battlefield trauma care in adults, has also been applied to neonates with NEC who present with severe preoperative physiological derangement in the context of extended disease. As described by Arul et al., the parameters included a median temperature of 35.5 °C, lactate of 3.7 mmol/L, an activated prothrombin time of 49 s, and dependence on a median of one inotrope (range 0–4). The approach involves resection of non-viable bowel, temporary ligation of the bowel ends, and leaving the abdomen open for planned subsequent procedures [127]. A subsequent study by the same author demonstrated that implementation of a regional damage-control surgery pathway led to reductions in multiple time metrics, including referral-to-PICU interval, PICU-to-surgery interval, and overall operative duration. Clinical outcomes, such as mortality, duration of parenteral nutrition, and surgical complication rates, were also improved compared with expectations based on historical datasets [128].

## 8. Postoperative Management of S-NEC

### 8.1. Prevention, Early Detection, and Management of Postoperative Complications

Infants undergoing surgery for NEC remain at exceptionally high risk for postoperative complications due to the severity of systemic illness, ongoing inflammation, and the physiological fragility of extreme prematurity. Postoperative care therefore centres on stabilising physiology, re-establishing nutrition, and identifying complications early—each of which directly impacts survival and long-term outcome (Table 2).

#### 8.1.1. Pain Control and Prevention of Physiological Instability

Uncontrolled pain can trigger significant haemodynamic and respiratory instability and contributes to adverse neurodevelopmental outcomes [129]. A structured pain assessment strategy is essential. Non-pharmacologic measures should be routine, with paracetamol as the first-line therapy and opioids reserved for severe pain. Excessive opioid exposure increases the risk of apnoea, intraventricular haemorrhage (IVH), and periventricular leukomalacia (PVL) [129,130]. Non-steroidal anti-inflammatory drugs (NSAIDs), particularly ketorolac, are avoided due to risks of bleeding and renal impairment [130].

#### 8.1.2. Fluid, Electrolyte, and Haemodynamic Complications: Circulatory Support and Transfusion

Haemodynamic instability is common in neonates with surgical NEC, and appropriate management is critical for optimising outcomes.

Fluid resuscitation with crystalloids remains the cornerstone of initial haemodynamic stabilisation in the acute post-surgical phase; however, no specific fluid resuscitation regimens can be universally recommended due to the limited quality of available evidence, which comes primarily from small, single-site studies [133].

The Society of Enhanced Recovery After Surgery (ERAS) recommendations for neonatal perioperative care emphasise that neonates require careful titration of intravenous fluid intake, particularly in the first few days of life [133]. Under-resuscitation risks hypoperfusion, while excessive fluids exacerbate intestinal oedema, impair anastomotic healing, and prolong ileus [131,132,133]. Fluid choice should be driven by the infant’s preoperative fluid status, intraoperative losses, urine output and electrolyte balance, with hypotonic solutions cautiously used due to the risk of hyponatremia [133]. Infants with ostomies are especially vulnerable to sodium depletion, and routine urinary sodium monitoring is recommended, with targets > 30 mmol/L [133].

The American Heart Association and American Academy of Pediatrics 2025 guidelines for neonatal resuscitation recommend normal saline (0.9% sodium chloride) as the crystalloid fluid of choice for volume expansion in newborn infants with circulatory compromise [172]. When blood loss is suspected in a newborn infant who responds poorly to resuscitation, administering a volume expander without delay may improve circulation and perfusion; uncrossmatched type O Rh-negative blood (or crossmatched, if immediately available) is preferred when blood loss is substantial [172].

Red blood cell (RBC) transfusion should be reserved for clinically significant anaemia or substantial blood loss and not used as a primary strategy for haemodynamic stabilisation. A 2024 international guideline recommends a restrictive transfusion strategy for preterm neonates born before 30 weeks’ gestation, with haemoglobin thresholds adjusted for postnatal age and respiratory support [135]: 11 g/dL (with respiratory support) or 10 g/dL (minimal/no support) in week 1; 10 g/dL or 8.5 g/dL in week 2; and 9 g/dL or 7 g/dL from week 3 onward. Respiratory support includes mechanical ventilation, continuous positive airway pressure, FiO_2_ > 0.35, or nasal cannula flow ≥ 1 L/min [173]. Large randomised trials, including the TOP and ETTNO studies (>3400 preterm infants), found no difference in death or neurodevelopmental impairment at 2 years between higher and lower transfusion thresholds, supporting restrictive transfusion practices [173,174]. Restrictive transfusion thresholds based on gestational age, respiratory support, and postnatal age are now favoured to avoid transfusion-associated morbidity [134,135], and phlebotomy losses should be minimised [136].

Both severe anaemia and RBC transfusion have been associated with increased NEC risk [7]. Observational data suggest that withholding enteral feeds during RBC transfusion may reduce transfusion-associated NEC, with a meta-analysis demonstrating a lower incidence when feeds were paused; however, randomised trial evidence is insufficient, and practice remains variable [30,175].

#### 8.1.3. Infectious Complications and Antimicrobial Management

Sepsis remains one of the most common and lethal postoperative complications. Empiric antibiotic coverage typically includes ampicillin, gentamicin, and metronidazole [137]. Prolonged antibiotic courses (>5 days) have not shown improved outcomes [138] yet are frequently used due to the high mortality of neonatal sepsis and concerns regarding immune immaturity. Overuse contributes to microbiome disruption, with negative downstream effects on gut barrier function [176].

#### 8.1.4. Nutritional Complications and Reintroduction of Enteral Feeding

While parenteral nutrition (PN) is essential for postoperative stabilisation, prolonged dependence carries substantial risk, including central-line-associated bloodstream infection (CLABSI) and cholestasis [139]. These complications are major contributors to impaired growth, neurodevelopmental delay, extended hospitalisation, and mortality [140].

Intestinal-failure-associated liver disease (IFALD) remains a frequent and potentially life-threatening complication of long-term PN following surgical necrotising enterocolitis (NEC), although outcomes have improved with advances in lipid emulsions and catheter care [177,178,179]. IFALD has a multifactorial pathogenesis involving hepatotoxic PN lipid components, gut dysbiosis with gut–liver axis activation, impaired intestinal barrier function, and sepsis, leading to cholestasis and progressive liver injury [178]. Early achievement of full enteral nutrition and timely discontinuation of PN remain the most effective preventive and therapeutic strategies, with structured feeding protocols shown to reduce PN duration and IFALD risk [177,179,180]. Modification of intravenous lipid emulsions is central to management: fish-oil-based emulsions, alone or within mixed formulations, can reverse cholestasis in most affected infants, whereas soy-based lipid emulsions are associated with a higher risk of IFALD [177].

The optimal strategy for reintroducing enteral nutrition after S-NEC remains unclear, with practice largely driven by expert consensus and institutional protocols rather than high-quality evidence [141,181]. Nevertheless, several consistent principles are supported by the available literature.

Mother’s own milk (MOM) is the preferred first choice when restarting feeds after NEC or focal intestinal perforation, based on expert consensus and physiological benefit [141]. Breast milk is strongly recommended as first-line enteral nutrition for both preterm and term infants, supported by high-quality evidence demonstrating reduced rates of NEC, late-onset sepsis, and other neonatal morbidities, such as chronic lung disease, ROP and neurodevelopmental impairment [133]. When MOM is unavailable, pasteurised donor human milk (PDHM) is recommended, as it significantly reduces NEC risk compared with formula, although it does not provide all the benefits of maternal milk [141]. When human milk cannot be provided, amino-acid-based formulas appear to be associated with better outcomes than hydrolysed formulas, particularly in infants with intestinal failure [180].

Early enteral nutrition (within 5–7 days postoperatively) has been associated with reduced rates of intestinal strictures, fewer reoperations, shorter hospital stay, and decreased duration of PN compared with delayed feeding [142]. Clinical and radiological parameters have been shown to help identify infants likely to tolerate early feeding following surgical NEC [182]. Importantly, there is no evidence supporting a mandatory 7-day period of nil per os after laparotomy for NEC, and early feed reintroduction appears safe in selected cases [183]. Prolonged postoperative gut rest has not demonstrated benefit and may delay enteral autonomy [180].

More rapid advancement of feed volumes (≥20 mL/kg/day) is associated with shorter hospitalisation and reduced parenteral nutrition exposure, without increased rates of NEC recurrence or stricture formation [142]. Combined continuous and bolus feeding strategies may further support intestinal adaptation and oral feeding development [180].

Early initiation of enteral feeding—within 7 days when feasible—is now supported as safe and is associated with fewer strictures, fewer reoperations, and shorter PN duration [15,142,183].

#### 8.1.5. Feeding Intolerance

Feeding intolerance is common during the reintroduction of enteral feeds and manifests as abdominal distention, vomiting, altered gastric residuals, or stool changes. Its pathophysiology likely reflects impaired motility, systemic inflammation, and gut dysbiosis. These infants often require prolonged respiratory and nutritional support and experience higher rates of ROP, septic shock, IFALD, and IVH, although these relationships are strongly influenced by underlying illness severity [143].

#### 8.1.6. Post-NEC Intestinal Stricture: Risk Factors and Management Strategies

Post-NEC intestinal stricture is the most frequent late complication. Despite acute NEC often involving the terminal ileum, up to 80% of strictures develop in the left colon [124]. Contributing factors include ischemic injury during the acute episode, intraoperative manipulation, haemodynamic instability, and absence of luminal flow distal to a stoma [145]. Early restoration of bowel continuity may reduce stricture formation [124,140]. The timing of stoma closure in NEC remains controversial. Although early closure (<8 weeks) and body weight < 2 kg are linked to prolonged postoperative hospitalisation, they do not seem to raise the risk of major surgical complications or mortality [146]. Limited evidence suggests mucous fistula refeeding can promote growth, shorten PN duration, and decrease cholestasis and anastomotic complications [133].

#### 8.1.7. Overall Morbidity Burden

A recent multicentre Dutch cohort of 326 infants with S-NEC reported an overall postoperative complication rate of 57.1% [144]. Major complications (63.4%) included anastomotic leak, ongoing NEC, adhesive obstruction, abscesses, compartment syndrome, multi-organ dysfunction, respiratory or renal failure, neurologic deterioration, and death. Sepsis (19.4%), stoma complications (13.3%), and wound dehiscence (11.3%) were the most common. Among stoma complications, stenosis is the most frequent cause, followed by high-output stoma, ileus, prolapse, and hernias [115].

Cardiovascular support prior to surgery predicted major complications, while stoma creation correlated with minor complications—likely reflecting selection bias toward more unstable infants [144].

#### 8.1.8. Predictors of Adverse Outcomes

Additional studies have identified antenatal steroid exposure as protective against postoperative complications, while jejunostomy increases risk [170]. Acute kidney injury after NEC onset is strongly associated with multiple postoperative complications. Predictive models for post-NEC stenosis incorporate admission weight, haematochezia, duration of CRP elevation, lactate levels, disappearance of intestinal peristalsis, and operative factors such as technique and duration [145].

### 8.2. Long-Term Outcomes in S-NEC

#### 8.2.1. Intestinal Failure and Nutritional Complications

Beyond these acute postoperative issues, S-NEC contributes substantially to long-term sequelae, such as IF, largely due to SBS and the reduction in functional absorptive surface [155]. IF can lead to diverse complications including IF-associated liver disease, as well as complications affecting the gastrointestinal tract, the kidneys and the skeletal system. Malabsorption may result in diarrhoea, micronutrient deficiencies, metabolic bone disease, renal impairment and inadequate growth. Device-related issues, such as central line infections and gastrostomy tube malposition, are also common in this population [149,150].

Risk factors for developing intestinal failure after surgical NEC include low birth weight, longer bowel loss, a higher percentage of small bowel resected and postoperative ileus days [147,148].

Nevertheless, the literature consistently indicates that most infants who survive surgical NEC eventually attain enteral autonomy [151]. This outcome is more likely when care is delivered through a multidisciplinary intestinal rehabilitation programme [152,155] and when surgical management emphasises maximal preservation of viable bowel. Predictors of achieving enteral autonomy include longer residual small bowel length, presence of the ileocecal valve, residual colon length which can partially compensate for reduced small bowel length, absence of stoma and fewer episodes of sepsis [152,153,154].

Despite favourable gastrointestinal outcomes, intestinal failure in paediatric patients has been associated with an increased risk of neurodevelopmental impairment, including deficits in cognitive, motor, and language domains [184,185,186,187]. These considerations emphasise that survival and enteral autonomy, while critical milestones, do not fully capture long-term morbidity, and neurodevelopmental outcomes should be considered an integral component of follow-up in this population.

#### 8.2.2. Neurodevelopmental Outcomes and the Gut–Brain Axis

As mentioned above, long-term outcomes extend beyond gastrointestinal recovery to include neurodevelopmental impairment (NDI), cerebral palsy, cognitive deficits, motor delays, and increased risk of white matter brain injury (WMBI) and periventricular leukomalacia (PVL) [156,157,158]. The incidence of NDI in S-NEC survivors is significantly higher than in medically managed NEC or preterm controls, with cognitive and motor deficits persisting into school age [156,158,188].

Patient-related factors such as lower gestational age and birth weight are strongly associated with worse neurodevelopmental outcomes [159,160]. Treatment-related factors, theoretically modifiable, including the use of peritoneal drains and enterostomies (as opposed to primary anastomosis), are linked to poorer neurological outcomes, likely due to ongoing inflammation and delayed restoration of gut integrity [159,161]. Additional risks—such as assisted mechanical ventilation, postoperative acute kidney injury, longer time to surgery, and cholestasis—further increase the likelihood of neurological, motor, and cognitive deficits [160,162]. Postoperative sepsis and retention of necrotic bowel are independent predictors of brain injury [162].

The gut–brain axis plays a central role in the pathogenesis of these complications. NEC-induced dysbiosis and systemic inflammation disrupt the immature blood–brain barrier, leading to microglial activation and neuroinflammation, which contribute to brain injury and NDI [161,163,164,165]. Alterations in the microbiome and persistent inflammatory signalling are implicated in adverse neurodevelopmental outcomes. Experimental evidence suggests that anti-inflammatory treatments targeting microglia may mitigate brain injury, but clinical application remains investigational [163,164,165].

Combined exposures, such as NEC and patent ductus arteriosus (PDA), synergistically impair cerebral development and increase the incidence of white matter injury and bloodstream infections, compounding neurodevelopmental risk [157,189]. Early recognition, prompt surgical intervention, and strategies to minimise systemic inflammation are critical to improving long-term outcomes in S-NEC. The European Reference Network recommends that the risk of neurodevelopmental disability and reduced quality of life should be taken into account during parental counselling, and emphasises that good clinical practice includes close follow-up of these patients [15].

#### 8.2.3. Pulmonary Outcomes and the Gut–Lung Axis

The gut–lung interaction plays a critical role in shaping long-term outcomes. The gut–lung axis is modulated by microbial metabolites and immune cell trafficking, with intestinal inflammation and barrier dysfunction promoting systemic immune activation that impairs pulmonary development and function [19]. These mechanisms may help explain the high burden of pulmonary morbidity in S-NEC, such as prolonged mechanical ventilation and severe bronchopulmonary dysplasia (BPD) [190]. Laparotomy provides more effective control of peritoneal contamination compared to peritoneal drainage, which is associated with worse respiratory outcomes and increased need for ongoing respiratory support [190].

Several clinical factors have been associated with the development and severity of BPD in the S-NEC population. Development of moderate-to-severe BPD is strongly associated with the presence of a patent ductus arteriosus, acute kidney injury, intestinal failure, poor linear growth, greater respiratory support requirements, and longer hospitalisations [166]. These comorbidities contribute to adverse neurodevelopmental outcomes and increased morbidity. Early identification of infants at risk and implementation of lung-protective strategies are essential to mitigate the burden of pulmonary morbidity in S-NEC [1,166].

#### 8.2.4. Ophthalmologic Sequelae: The Gut–Retina Axis

S-NEC has been linked to severe retinopathy of prematurity (ROP), largely because the two conditions share several major risk factors and pathophysiological pathways, including extreme prematurity, prolonged oxygen exposure, systemic inflammatory response, and sepsis [167,168,169]. Elevated inflammatory cytokines and oxidative stress can disrupt normal retinal vascular development, while haemodynamic and respiratory instability further contribute to retinal ischemia and aberrant neovascularisation [168,169]. Emerging evidence also suggests that gut dysbiosis may adversely influence retinal angiogenesis by altering key mediators such as vascular endothelial growth factor (VEGF) and insulin-like growth factor-1 (IGF-1) [168,171].

In summary, surgical NEC survivors face a high risk of impaired quality of life, especially in psychosocial domains, but many achieve near-normal physical functioning if major comorbidities are absent [191,192].

## 9. Gaps and Areas of Ongoing Research in S-NEC

NEC, and particularly its severe form (S-NEC), represents a life-defining diagnosis for survivors and their families and one of the most challenging conditions encountered in neonatal surgery. Its long-term impact extends across physical, neurodevelopmental, and psychosocial domains, while also imposing a substantial financial burden on healthcare systems [192]. Despite major advances in neonatal care, fundamental gaps persist in our understanding of NEC pathophysiology, progression, and optimal surgical management.

Key controversies continue to shape clinical decision-making in S-NEC, particularly regarding the indications and timing for operative intervention [86,87]. Accurate identification of infants who will ultimately require surgery remains difficult. Ongoing research is focused on improving early risk stratification through refinement of clinical scoring systems, evaluation of individual biomarkers such as faecal calprotectin, S100A12, and serum fatty-acid-binding protein, and incorporation of advanced imaging modalities, including targeted abdominal ultrasound and near-infrared spectroscopy, to support more timely and objective operative decision-making [44,58].

Although patient-specific factors must guide management, establishing general principles to standardise care is essential. High-quality prospective studies are needed to address these gaps, and emerging artificial intelligence applications in paediatric surgery may facilitate the development of predictive models capable of improving diagnostic accuracy and surgical timing [68,82].

Operative strategy for S-NEC also remains an area of active debate. Decisions regarding the extent of resection and the choice between primary anastomosis and stoma creation vary across institutions [113,118,193]. Current practice generally supports complete excision of clearly non-viable bowel, given the systemic impact of ongoing intra-abdominal sepsis on the gut–brain, gut–lung, and gut–retina axes [163,166,169]. Although concerns regarding short bowel syndrome and intestinal failure are substantial, many cases of NEC-related intestinal failure ultimately achieve enteral autonomy; however, access to specialised intestinal rehabilitation programmes remains inconsistent worldwide [154].

When anatomy and physiology permit, primary anastomosis is increasingly favoured [15], as accumulating evidence suggests that restoring bowel continuity does not increase postoperative complications in appropriately selected neonates. The selective use of laparoscopy is also being explored [92,98,99,108]. Proposed advantages include improved visualisation and a potential CO_2_-mediated anti-inflammatory effect [92], but laparoscopy is not yet widely adopted in this setting and cannot be considered standard practice [100,101].

## 10. Future Directions

Several emerging research avenues have broadened the landscape of potential preventive and therapeutic strategies for necrotising enterocolitis (NEC), although most remain experimental and are not ready for routine clinical or surgical application.

Remote ischemic conditioning (RIC) has strong preclinical support, demonstrating reduced intestinal injury [194] and improved microcirculation in NEC models [195,196]. Notably, the only available transcriptomic analysis has shown that RIC exerts intestinal protection not only by mitigating ischemic injury but also by modulating anti-inflammatory pathways [197], which is highly relevant given the multifactorial pathophysiology of NEC. Early clinical studies confirm feasibility and safety in preterm infants, and a multicentre phase II trial is underway [198,199].

Microbiota-based interventions continue to evolve. Faecal microbiota transplantation (FMT) reduces inflammation and mucosal injury in animal models but carries risks of pathogen transmission in neonates [200]. Faecal filtrate transplantation (FFT), which excludes bacteria but retains bacteriophages, has shown protection in piglet models, though human data remain absent [201].

Probiotics represent one of the most extensively studied preventive strategies for necrotising enterocolitis; however, their adoption remains variable across neonatal units. This variability largely reflects ongoing concerns regarding strain selection, regulatory oversight, and safety [202]. Recent meta-analyses suggest that multi-strain formulations, particularly when combined with oligosaccharides, are associated with a significant reduction in the incidence of severe NEC and overall mortality in very preterm infants [203].

These findings are further supported by a large retrospective cohort study of 48,048 infants born before 32 weeks’ gestation, which reported associations between probiotic exposure and lower rates of severe NEC, reduced late-onset sepsis, and improved survival to discharge [202]. In addition, strain-specific effects have been suggested by a single-centre retrospective study demonstrating lower NEC rates during routine administration of *Bifidobacterium infantis* (EVC001), compared with periods before its introduction and after discontinuation [204].

Nevertheless, conclusions regarding probiotic efficacy are not entirely uniform, as reflected in the most recent Cochrane review, which may partly explain the continued hesitancy among some neonatal units to implement routine probiotic supplementation [35].

Probiotics are the most clinically advanced strategy. Meta-analyses demonstrate that multi-strain formulations, especially when paired with oligosaccharides, significantly reduce severe NEC and mortality in very preterm infants [35,203]. Optimal strains, dosing, and safety in the most immature neonates remain under investigation.

Stem cell therapies—including mesenchymal stromal cells (MSCs) and amniotic fluid stem cells (AFSCs)—consistently decrease NEC severity in rodent models, with preliminary evidence favouring AFSCs [205,206]. Translation to clinical practice will require rigorous safety and dosing trials.

Exosome-based treatments from breast milk or stem cells appear to strengthen barrier function and blunt inflammation in experimental NEC, but these approaches remain confined to laboratory research [207,208].

Targeted immunomodulatory therapies aimed at pathways such as TLR signalling and type 3 cytokines, including IL-37, have shown benefit in murine models, though human data remain sparse [209].

Overall, probiotics are the only emerging modality with demonstrated clinical benefit in NEC prevention. Other strategies, including RIC, FMT/FFT, stem cell and exosome therapies, and targeted immunomodulation, are promising but require further translational evaluation before integration into surgical care.

## 11. Conclusions

In summary, operative management of NEC remains one of the most challenging decisions in neonatal surgery. Each intervention must be tailored to the extent of abdominal disease, the infant’s physiological reserve, the surgeon’s technical judgment, and the resources of the treating centre. Emerging evidence makes one point unequivocal: timing is as critical as technique. When surgery is performed—before irreversible physiological collapse—has a decisive influence on survival, postoperative recovery, and long-term outcomes.

As surgical strategies advance, a precise understanding of the downstream complications of NEC is no longer optional—it is fundamental to optimising care pathways and providing clear, honest counselling to families facing a life-defining diagnosis. The field now needs more than incremental progress. It demands rigorous external validation of predictive tools, coordinated multicentre prospective studies, and translational research capable of delivering truly disease-modifying therapies.

Equally urgent is the need for consensus: a unified definition of clinical deterioration despite maximal medical therapy, and the establishment of a standardised, evidence-based European feeding protocol for infants recovering from NEC. These efforts are essential steps toward improving outcomes for the most vulnerable patients in paediatric surgery.

## Figures and Tables

**Table 1:** S-NEC predictors. Abbreviations: S-NEC, surgical necrotising enterocolitis; AUC, area under the receiver operating characteristic curve; CRP, C-reactive protein; PLR, platelet-to-lymphocyte ratio; i-FABP, intestinal fatty-acid-binding protein; X-ray, plain radiographic imaging; US, ultrasound; MD7, metabolic derangement score; AI, artificial intelligence; DL, deep learning.

Category	Key Predictors
**Clinical Predictors**	Serial physical examination (most reliable predictor); Physical exam score ≥ 3: sensitivity 0.88, specificity 0.81, AUC 0.89 [43]; Abdominal distention (high sensitivity) [44]; Abdominal wall erythema (high specificity) [44].
**Laboratory/Biomarkers**	CRP elevation [40,45,46,47,48,49,50]; CRP/albumin ratio [51,52]; High procalcitonin [45,49,53]; Acidosis [36,39,50,54]; Thrombocytopenia [46,50], high MPV/PDW [55]; PLR [45], lymphopenia [46,47,50], neutropenia [45,54,56]; Coagulation disorder [40,57]; Hyponatremia [40,50], hypoalbuminemia [48]; i-FABP (highly specific but not practical) [49,58].
**Diagnostic Paracentesis**	Positive peritoneal tap (≥0.5 mL cloudy/clear fluid + Gram stain) [59,60]; Combined with thrombocytopenia, neutropenia, hyponatremia: strong predictors [17]; Routinely used by 76.3% of paediatric surgeons in China [61].
**Abdominal Radiography**	Pneumoperitoneum = only absolute surgical indication; Signs confirming perforation (supine X-ray): liver shadow lucency, falciform ligament sign, Rigler sign, football sign, triangle sign [62]; Sensitivity 86–73%, specificity 100% [62]; Portal venous gas, fixed loops: high specificity, low sensitivity [44]; Increased bowel wall diameter relative to vertebral landmarks [63]; Limitations: misses ~50% of perforations, radiation exposure [64].
**Abdominal Ultrasound**	Thinned bowel wall [65,66]; Absent peristalsis [65,66]; Complex ascites [65,66]; Pneumoperitoneum [65]; Predicting resection: thick → thin transition, absent perfusion, portal venous gas [67]; In 25% of cases with radiographic pneumoperitoneum, there was a lack of US evidence of perforation [64]; US + X-ray combined improves diagnostic accuracy [64].
**Scoring Systems**	MD7 for infants without pneumoperitoneum, most frequently used, specificity and sensitivity > 70%, threshold ≥ 3 [58].
**AI and DL**	ResNet18 model [68]; CatBoost algorithm [69].

**Table 2:** Surgical NEC (S-NEC) postoperative complications and short- and long-term outcomes. Abbreviations: NSAIDs, non-steroidal anti-inflammatory drugs; IVH, intraventricular haemorrhage; PVL, periventricular leukomalacia; PN, parenteral nutrition; CLABSI, central-line-associated bloodstream infection; LOS, length of hospital stay; HM, human milk; MOM, mother’s own milk; ACS, abdominal compartment syndrome; GI, gastrointestinal; SBS, short bowel syndrome; BW, body weight; IFALD, intestinal-failure-associated liver disease; ICV, ileocecal valve; NDI, neurodevelopmental impairment; GA, gestational age; AKI, acute kidney injury; BPD, bronchopulmonary disease; PDA, patent ductus arteriosus; IF, intestinal failure; PPD, primary peritoneal drainage; VEGF/IGF-1, vascular endothelial growth factor/insulin-like growth factor-1; ROP, retinopathy of prematurity.

Domain	Key Elements	Summary
**Physiological Support**	*Pain*	Paracetamol first-line; opioids for breakthrough. Avoid NSAIDs. Morphine ↑ apnoea/IVH/PVL risk [129,130].
	*Fluids*	Avoid hypo/hypervolemia; monitor electrolytes. Urine Na > 30 mmol/L; ↑ losses in ostomy patients [131,132,133].
	*Blood Products*	Restrictive thresholds: 11 → 10 → 9 g/dL (respiratory support); 10 → 8.5 → 7 g/dL (minimal support). Minimise phlebotomy [134,135,136].
	*Infection*	Ampicillin + gentamicin ± metronidazole. Usually 7–14 days; >5 days not shown to be beneficial [137,138].
**Nutrition**	*Parenteral Nutrition*	Early PN essential; risks: CLABSI, cholestasis [139], impaired growth, prolonged LOS [140].
	*Enteral Feeding*	Start ≤7 days → ↓ strictures, PN duration, LOS [15]. Prefer HM [136]; MOM is the preferred first choice [141].
	*Protocols*	Structured advancement improves consistency; ≥20 mL/kg/day may shorten LOS [142].
	*Feeding Intolerance*	From inflammation, dysmotility, dysbiosis; associated with illness severity [143].
**Postoperative Complications**	*Early GI*	Major (63.4%): Abscess, adhesions, ACS, leakage. Mucous fistula refeeding ↓ PN time and cholestasis. Stoma-related 13.3% (high output, prolapse, stenosis, hernia). Wound dehiscence 11.3%. Risk factors: cardiovascular support, enterostomy [144].
	*Strictures*	Most common; 80% left colon [124]; ischemia/diversion-related [145]. Early continuity may reduce [124,140,146].
	*Other*	Sepsis 19.4%, respiratory or renal failure, neurologic deterioration [144].
**Long-Term GI Outcomes**	*Intestinal Failure/SBS*	From bowel loss. Risks: low BW, major resection, ileus [147,148]. CLABSI, gastrostomy tube dislocation, IFALD common [149,150].
	*Enteral Autonomy*	Most achieve autonomy [151]. Predictors: residual bowel length, ICV, colon, no stoma, ↓ sepsis, rehabilitation programme [152,153,154,155].
**Neurodevelopment**	*NDI and Brain Injury*	High rates: WMBI, PVL, CP [156,157,158]. Risk factors: low GA and BW [159,160], drains, enterostomies [159,161], AKI, sepsis, cholestasis [160,162].
	*Mechanisms*	Dysbiosis → systemic inflammation → microglial activation → neuroinflammation [161,163,164,165].
**Respiratory**	*Pulmonary Morbidity*	BPD risk factors: PDA, AKI, IF, prolonged ventilation, PPD vs. laparotomy [166].
	*Mechanisms*	Gut–lung axis: inflammation and barrier dysfunction → lung injury [19].
**Ophthalmology**	*ROP*	Related to prematurity, inflammation, oxygen exposure [167,168,169]; microbiome influences VEGF/IGF-1 [170,171].

## Data Availability

No new data were created or analysed in this study.

## Abbreviations

The following abbreviations are used in this manuscript:

NEC	Necrotising Enterocolitis
S-NEC	Surgical Necrotising Enterocolitis
VLBW	Very Low Birth Weight
NICU	Neonatal Intensive Unit
TANEC	Transfusion-Associated NEC
FIP/SIP	Focal Intestinal Perforation
FPIES	Food-Protein-Induced Enterocolitis
ELBW	Extremely Low Birth Weight
TLR4	Toll-Like Receptor-4
IUGR	Intrauterine Growth Restriction
ICP	Intrahepatic Cholestasis of Pregnancy
MSAF	Meconium-stained Amniotic Fluid
PDA	Patent Ductus Arteriosus
SGA	Small for Gestational Age
IVH	Intraventricular Haemorrhage
NSAIDs	Non-Steroidal Anti-Inflammatory Drugs
AUC	Arterial Umbilical Catheter
VUC	Venous Umbilical Catheter
AI	Artificial Intelligence
DL/ML	Deep Learning/Machine Learning
MPV	Mean Platelet Volume
PDW	Platelet Distribution Width
PLR	Platelet-to-Lymphocyte Ratio
I-FABP	Intestinal Fatty Acid Binding Protein
MD7	Metabolic Derangement 7 score
PPD/PD	Primary Peritoneal Drainage/Peritoneal Drain
GA	Gestational Age
BW	Body Weight
LOS	Length of Hospital Stay
AKI	Acute Kidney Injury
PN/TPN	Parenteral Nutrition/Total Parenteral Nutrition
CLABSI	Central-Line-Associated Bloodstream Infections
HM	Human Milk
ACS	Abdominal Compartment Syndrome
BPD	Bronchopulmonary Dysplasia
RCTs	Randomised Controlled Trials
ICG	Indocyanine Green
DCS	Damage-Control Surgery
PICU	Paediatric Intensive Care Unit
PVL	Periventricular Leukomalacia
CP	Cerebral Palsy
ROP	Retinopathy of Prematurity
VEGF	Vascular Endothelial Growth Factor
IGF-1	Insulin Growth Factor-1
IFALD	Intestinal-Failure-Associated Liver Disease
IF	Intestinal Failure
ICV	Ileocecal Valve
SBS	Small Bowel Syndrome
NDI	Neurodevelopmental Impairment
WMBI	White Matter Brain Injury
RIC	Remote Ischemic Conditioning
FMT	Faecal Microbiota Transplantation
FFT	Faecal Filtrate Transplantation
MSCs	Mesenchymal Stem Cells
AFSCs	Amniotic Fluid Stem Cells
